# Repair Kinetics of DNA Double Strand Breaks Induced by Simulated Space Radiation

**DOI:** 10.3390/life10120341

**Published:** 2020-12-10

**Authors:** Takashi Oizumi, Rieko Ohno, Souichiro Yamabe, Tomoo Funayama, Asako J. Nakamura

**Affiliations:** 1 Department of Biological Sciences, College of Science, Ibaraki University, Mito, Ibaraki 310-8512, Japan; 19nd101n@vc.ibaraki.ac.jp (T.O.); 17s4007f@vc.ibaraki.ac.jp (R.O.); 5010509096@edu.k.u-tokyo.ac.jp (S.Y.); 2 Department of Radiation-Applied Biology Research, Takasaki Advanced Radiation Research Institute, National Institutes for Quantum and Radiological Science and Technology (QST), Takasaki, Gunma 370-1292, Japan; funayama.tomo@qst.go.jp

**Keywords:** space radiation, DNA double strand break, DNA repair, heavy ion, linear energy transfer

## Abstract

Radiation is unavoidable in space. Energetic particles in space radiation are reported to induce cluster DNA damage that is difficult to repair. In this study, normal human fibroblasts were irradiated with components of space radiation such as proton, helium, or carbon ion beams. Immunostaining for γ-H2AX and 53BP1 was performed over time to evaluate the kinetics of DNA damage repair. Our data clearly show that the repair kinetics of DNA double strand breaks (DSBs) induced by carbon ion irradiation, which has a high linear energy transfer (LET), are significantly slower than those of proton and helium ion irradiation. Mixed irradiation with carbon ions, followed by helium ions, did not have an additive effect on the DSB repair kinetics. Interestingly, the mean γ-H2AX focus size was shown to increase with LET, suggesting that the delay in repair kinetics was due to damage that is more complex. Further, the 53BP1 focus size also increased in an LET-dependent manner. Repair of DSBs, characterized by large 53BP1 foci, was a slow process within the biphasic kinetics of DSB repair, suggesting non-homologous end joining with error-prone end resection. Our data suggest that the biological effects of space radiation may be significantly influenced by the dose as well as the type of radiation exposure.

## 1. Introduction

Exposure to ionizing radiation is one of the problems associated with human activities in space. The average annual dose of ionizing radiation on the ground is 2.4 mSv, whereas the average exposure on the International Space Station (ISS) is 0.5–1 mSv per day [1]. In addition, astronauts experience exposure of 1–2 mSv/day in deep space, and the cumulative effective radiation dose for a Mars mission is estimated to be as high as 1 Sv [2]. Further, space radiation comprises of galactic cosmic rays (GCRs) and solar particle events (SPEs), which contain approximately 85% protons, 14% helium ions, and 1% high-energy heavy ions (known as HZE particles) [3]. Heavy ion beams with high linear energy transfer (LET) in space radiation are known to cause cluster DNA damage in which multiple DNA damage occurs within 1–2 helix turns of a DNA molecule [4,5,6,7]. Compared to X-rays or γ-rays, heavy ion beams cause more chromosomal aberrations such as dicentric chromosomes, translocations, and deletion mutations, resulting in significant biological effects [8,9,10,11,12,13,14,15].

DNA double strand breaks (DSBs), the most severe ionizing radiation induced DNA damage, are repaired by canonical non-homologous end joining (c-NHEJ) or homologous recombination (HR) [16,17]. DSB repair by c-NHEJ occurs throughout the cell cycle, whereas the HR pathway functions only during the S and G2 phases, because it requires sister chromatids [18,19]. When a DSB is introduced, kinases such as ATM are activated immediately and they phosphorylate H2AX, a variant of histone H2A, around the DSB [20,21]. Following the formation of phosphorylated H2AX (γ-H2AX), 53BP1 is recruited to the DSB site. 53BP1 has an important role in the selection of the DSBs repair pathway [22,23,24,25].

Elucidation of the detailed mechanism underlying the DSB induction and repair processes after simulated space radiation exposure is critically important to evaluate the biological impact of space radiation. Therefore, in this study, we focused on the repair of DSBs induced in normal human fibroblasts by proton, helium ion, or carbon ion beams. Furthermore, using cocktail acceleration technology of Takasaki Ion Accelerators for Advanced Radiation Application (TIARA) cyclotron facility at QST-Takasaki that can switch between low-LET helium ions and high-LET carbon ions in a very short period of time, we report for the first time the effects of a mix of carbon and helium radiation on DNA damage repair.

## 2. Materials and Methods

### 2.1. Cell Culture

The human fetal lung fibroblast cell line, TIG-3-20, was purchased from JCBR cell bank, National Institutes of Biomedical Innovation, Health, and Nutrition. TIG-3-20 cells were cultured in Dulbecco’s modified Eagle’s medium (Wako, Osaka, Japan) containing 10% fetal bovine serum (173012, Sigma-Aldrich, St. Louis, MO, USA), 100 μg/mL penicillin and streptomycin (Wako) at 37 °C in a humidified atmosphere with 5% CO_2_. TIG-3-20 cells were used between passage 30 and 40.

### 2.2. Radiation Exposure

The cells were exposed to 1 Gy of monoenergetic 20-MeV proton, 63-MeV helium ion, and 190-MeV carbon ions accelerated using an AVF930 cyclotron (Sumitomo Heavy Industries, Tokyo, Japan) at TIARA of the Takasaki Advanced Radiation Research Institute (TARRI), National Institutes for Quantum and Radiological Science and Technology (QST) in Gunma, Japan. The mixed irradiation samples were irradiated with 0.5 Gy of carbon ion followed by 0.5 Gy of helium ion, and it took about 20–30 min to switch the beam. The LET value and dose rates of carbon ions on the cell surface were estimated as described previously [26], giving values of 2.73 keV/μm proton (4 Gy/min), 13.2 keV/μm helium ion (1.4 Gy/min), and 124 keV/μm carbon ion (3.4 Gy/min). The culture medium was temporarily removed from the irradiation vessels just before irradiation, and the vessels were capped with a thin sheet of polyimide film (Du Pont–Toray, Tokyo, Japan) to prevent the samples from drying out and being contaminated during irradiation at room temperature. Control cells were sham-irradiated and handled in parallel with the test cells.

### 2.3. Immunofluorescence Staining

Irradiated cells were fixed in 4% paraformaldehyde for 10 min at room temperature and permeabilized overnight with 70% ethanol at 4 °C. The cells were blocked with 4% bovine serum albumin, 0.5% Tween 20, and 0.1% Triton X-100 in PBS for 1 h at room temperature after hydrophilic treatment with PBS for 30 min at room temperature. Cells were then immunostained with the following antibodies: γ-H2AX (1:1000, 05-636, Millipore, Temecula, CA, USA) and 53BP1 (1:500, NB100-305, Novus Biologicals, Centennial, CO, USA). Secondary antibodies conjugated with Alexa488 (Thermo Fisher Scientific, Waltham, MA, USA) were then added and VECTASHIELD^®^ Antifade Mounting Medium with Propidium Iodide (Vector Laboratories, Burlingame, CA, USA) was used as a mounting agent. Fluorescence was detected using a fluorescence microscope (BX53, Olympus, Tokyo, Japan). γ-H2AX and 53BP1 foci were counted manually in 100 randomly chosen cells and were averaged across all counted cells in each experimental condition. Focus sizes were measured using ImageJ software.

### 2.4. Statistical Analysis

Differences in the number of γ-H2AX foci or 53BP1 foci between the irradiated groups were analyzed using Student’s t-test at each time point using Microsoft excel 2016. Kruskal–Wallis tests were performed to check the homogeneity distributions of the number of γ-H2AX foci in each cell and the size of foci using R version 4.0.2.

## 3. Results

### 3.1. DSB Repair Kinetics Depend on the Atomic Number of the Particle Beam

Although heavy ions account for only 1% of space radiation, their biological effects are considered significant because heavy ions induce complex damage in DNA [5,6,7]. In addition, because astronauts are exposed simultaneously to a wide range of energies and various ion species in space [27], the combined effects of exposure to different types of radiation must be considered. There are only a few irradiation facilities, which can be used to simulate radiations with ions of different energies and ion species, to study their effects on cells. The TIARA cyclotron facility at QST-Takasaki allows us to prepare cell samples that are irradiated with a mix of different ions almost simultaneously. Therefore, we decided to use this mixed radiation in our research. However, the ion species and energies of the ions that can be combined in mixed irradiation are limited. Thus, we chose a combination of helium and carbon, which can better simulate space radiation under limited conditions. For the proton irradiation, we used the same irradiation facility as for the mixed irradiation in order to maintain the same irradiation experiment conditions. Therefore, TIG-3-20 human fetal lung fibroblasts were irradiated with proton, helium, carbon ions, and helium ions irradiation after carbons ion irradiation as mixed irradiation at the TIARA cyclotron facility. In addition, irradiated cells were stained for the DSB marker γ-H2AX [20,21,28,29], to examine DSB levels.

**Figure 1:**
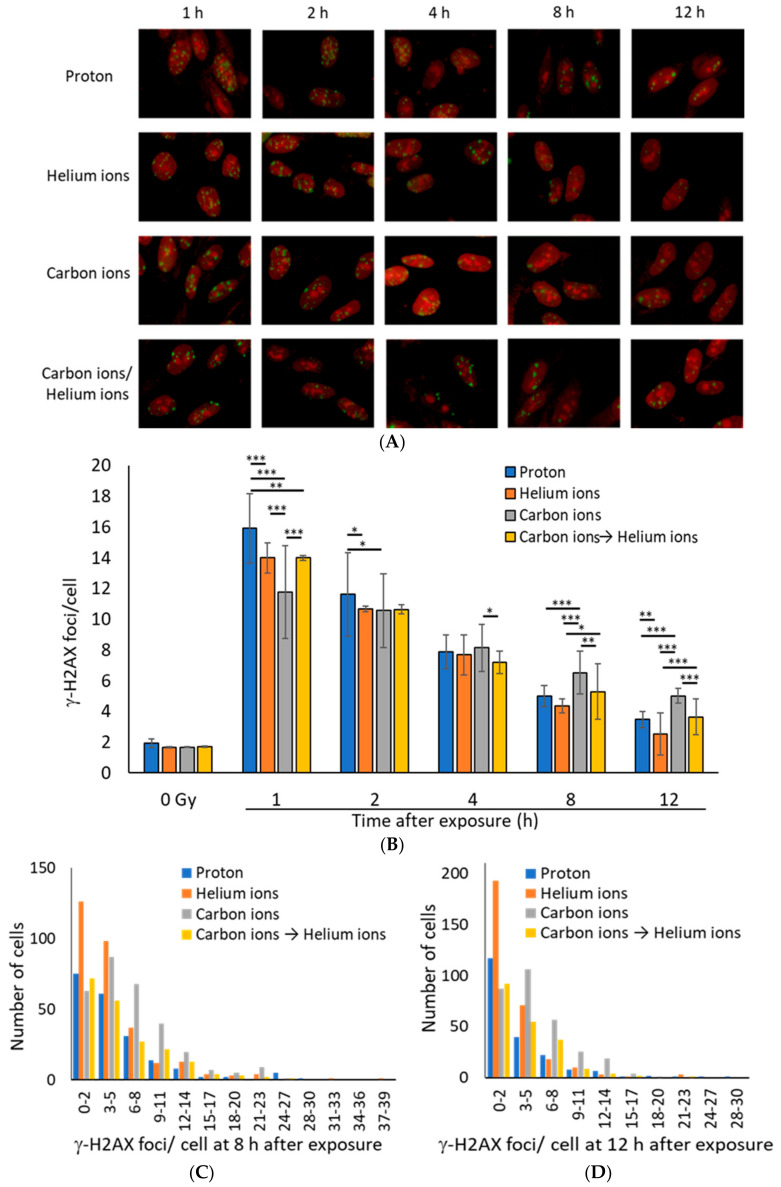
(**A**) Representative immunofluorescence images for γ-H2AX (green: γ-H2AX, Red: Propidium iodide). TIG-3-20 cells were irradiated with 1 Gy of monoenergetic 20-MeV protons (2.73 keV/μm), 63-MeV helium ions (13 keV/μm), and 190-MeV carbon ions (117 keV/μm). The mixed irradiation samples were irradiated with 0.5 Gy of carbon ions followed by 0.5 Gy of helium ions, with about 20–30 min required to switch the beam. (**B**) The mean number of γ-H2AX foci per cell after 1 Gy exposure. The data were obtained by counting the γ-H2AX foci for 100 cells (protons: *n* = 2, helium and carbon: *n* = 3, mixed carbon ion and helium ion: *n* = 2. Error bars: SD. *t*-test: * *p* < 0.05, ** *p* < 0.01, *** *p* < 0.001). (**C**,**D**) The histogram shows the number of γ-H2AX foci in each cell at 8 h (**C**) and 12 h (**D**) after irradiation. The histogram values were obtained from 200 proton irradiated cells, 300 helium ion irradiated cells, 300 carbon ion irradiated cells, 200 carbon and helium ion mix irradiated cells (Kruskal–Wallis test of proton vs. helium ions vs. carbon ions vs. carbon ions→helium ion: (**C**) *p*-value = 1.736 × 10^−10^, (D) *p*-value < 2.2 × 10^−16^).

At 1 h after irradiation, the average number of γ-H2AX foci per cell was the highest for proton (Z = 1) beams, followed by helium (Z = 2) beams, and was the lowest for carbon (Z = 6) beams (Figure 1A,B). Although DSB levels induced by carbon ion irradiation were seemingly, the lowest, accurate assessment of DSB levels is probably not possible. If the LET of a particle beam is high, the number of DSBs in a single γ-H2AX focus is likely to differ between beam types because of the high-density energy transfer to the range of a few microns [4,30,31]. In any type of radiation, the number of γ-H2AX foci decreased in 1–12 h after irradiation, indicating that DSB repair was underway (Figure 1B). However, the number of γ-H2AX foci in carbon-ion-irradiated cells at 8 or 12 h after irradiation was higher than that with proton or helium ion irradiation, suggesting that the repair kinetics of DSBs caused by carbon-irradiation were slow (Figure 1C,D).

Interestingly, the number of γ-H2AX foci in the mixed irradiation samples, which were irradiated with 0.5 Gy of carbon ions followed by 0.5 Gy of helium ions, was approximately equal to the average number of foci for the carbon and helium ion irradiation samples at any time point, suggesting that the mixed irradiation did not have any additive effect on the DSB repair kinetics (Figure 1B).

### 3.2. γ-H2AX Focus Size Depends on the LET of the Ion Beam

**Figure 2:**
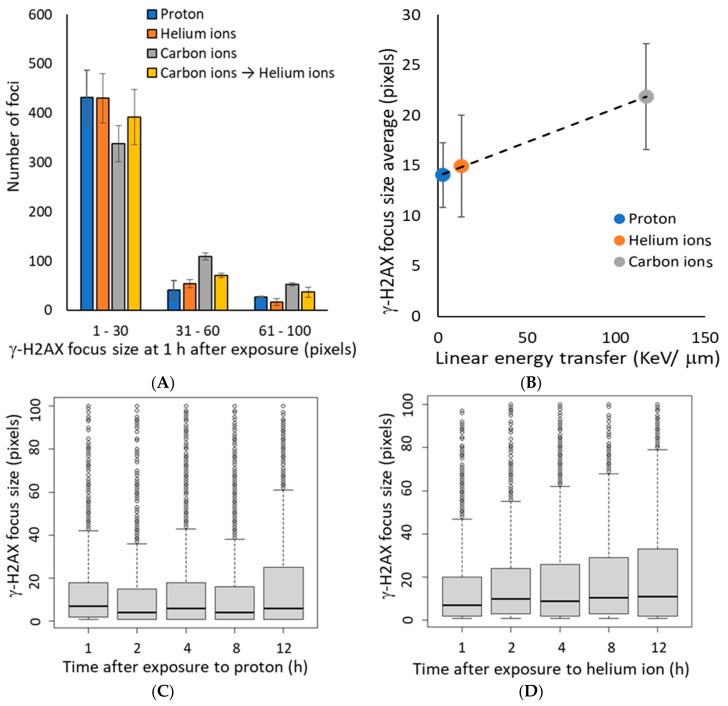

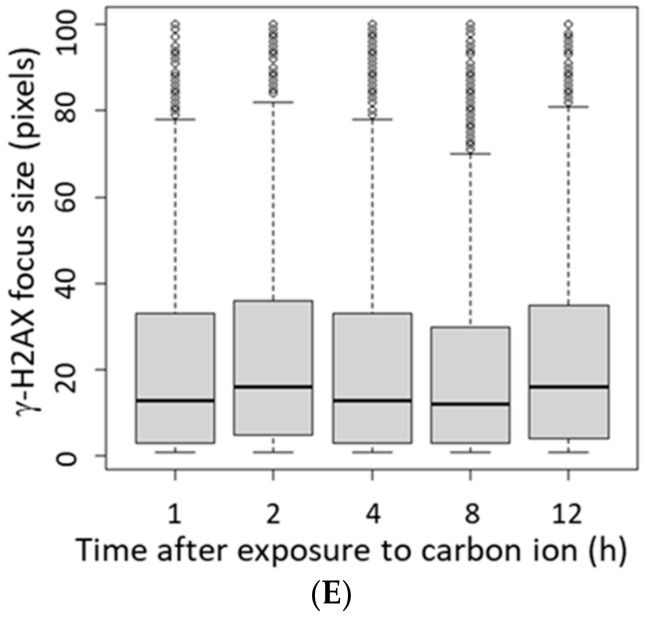
(**A**) The histogram shows the number of γ-H2AX foci defined by size at 1 h after irradiation. Focus size indicates the number of pixels calculated by image J for 500 γ-H2AX foci in each independent experiment (Kruskal–Wallis test of proton vs. helium ions vs. carbon ions vs. carbon ions→helium ion: *p*-value < 2.2 × 10^−16^). (**B**) The mean of average γ-H2AX focus size plotted by LET at 1 h after exposure. The dashed line shows the approximate line (R^2^ = 0.995). The LET 2.73 keV/μm for protons, 13 keV/μm for helium ions, and 117 keV/μm for carbon ions (protons: *n* = 2, helium and carbon: *n* = 3, mixed carbon ion and helium ion: *n* = 2, error bars: SD.). (**C**–**E**) The relationship between time after irradiation and change in the size of γ-H2AX foci is shown as a boxplot: (**C**) protons, (**D**) helium ions, (**E**) carbon ions. In the boxplot, the median of the lower half (first quartile) to the median of the upper half of the dataset (third quartile) is represented as a box, and the horizontal bar inside the box is the middle of the dataset. The vertical bars (also called whiskers) extending up and down from the box represent the maximum and minimum values included in the 1.5 times the interquartile range (IQR), and the dots represent outliers that are not included in the 1.5 times the IQR.

As the γ-H2AX focus size is reported to be large in regions with increasing number of DSBs [30], we analyzed the γ-H2AX focus size at 1 h after irradiation. The γ-H2AX focus size was positively correlated with the level of LET (Figure 2A,B), indicating that higher LETs were more likely a more number of DSBs per γ-H2AX focus. In this study, we categorized 30 pixels or less as small foci and 31 pixels or larger as large foci. We expected the average γ-H2AX focus size to increase with time after irradiation due to faster repair of small γ-H2AX foci, which represent relatively mild injury; however, the average γ-H2AX focus size did not change from 1–12 h post-irradiation, regardless of the irradiation type (Figure 2C–E). These data suggest that the large size of the γ-H2AX focus reduced gradually because of the slow progressive repair of multiple DSBs with cluster DNA damage, rather than the persistence of the damage.

### 3.3. 53BP1 Foci Associated with DSBs Induced by High LET Irradiation Undergo Slow Repair

**Figure 3:**
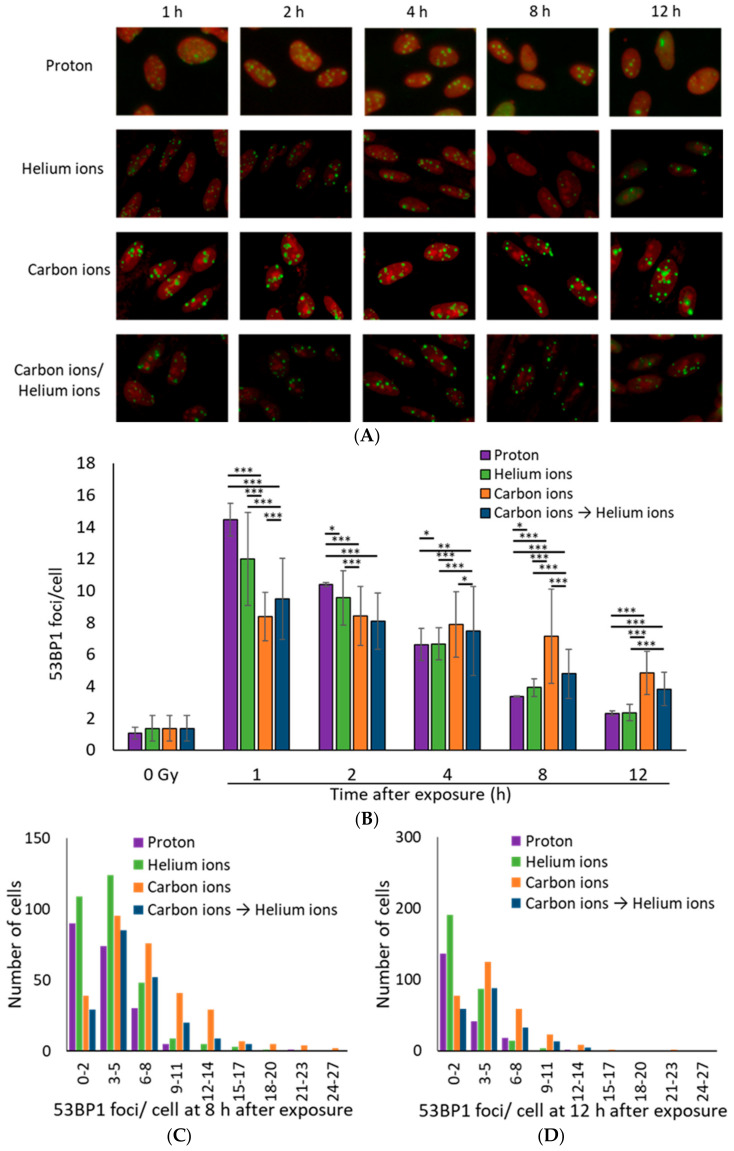
(**A**) Representative immunofluorescence images for 53BP1 (green: 53BP1, Red: Propidium iodide). TIG-3-20 cells were irradiated with 1 Gy of monoenergetic 20-MeV proton (2.73 keV/μm), 63-MeV helium ions (13 keV/μm), and 190-MeV carbon ions (117 keV/μm). The mixed irradiation samples were irradiated with 0.5 Gy of carbon ions followed by 0.5 Gy of helium ions, with about 20–30 min required to switch the beam. (**B**) The mean number of 53BP1 foci per cell after 1 Gy exposure. The data were calculated by counting 53BP1 foci in 100 cells (proton: *n* = 2, helium ions, carbon ions, and mixed carbon ions and helium ions: *n* = 3, respectively. Error bars: SD. t-test: * *p* < 0.05, ** *p* < 0.01, *** *p* < 0.001). (**C**,**D**) The histogram shows the number of 53BP1 foci in each cell at 8 h (**C**) and 12 h (**D**) after exposure. The histogram values were obtained from 200 proton irradiated cells, 300 helium ion irradiated cells, 300 carbon ion irradiated cells, 200 carbon and helium ion mix irradiated cells (Kruskal–Wallis test of proton vs. helium ions vs. carbon ions vs. carbon ions→helium ion: (**C**) *p*-value < 2.2 × 10^−16^, (**D**) *p*-value < 2.2 × 10^−16^).

53BP1, a DSB marker, is an important protein for the DSB repair process, which regulates switching of the DSB repair pathway by controlling DNA end resection [19,23,25]. Irradiated cells were thus stained with 53BP1 to examine DSB repair in more detail. Similar to the γ-H2AX foci, the average number of 53BP1 foci per cell was the highest for proton rays, followed by helium rays, and was the lowest for carbon rays at 1 h after irradiation (Figure 3A,B). Moreover, more 53BP1 foci remained in the carbon irradiated samples compared to the proton and helium irradiated samples at 12 h post irradiation (Figure 3C,D). These data support the slow DSB repair in carbon-irradiated samples seen in the γ-H2AX focus.

**Figure 4:**
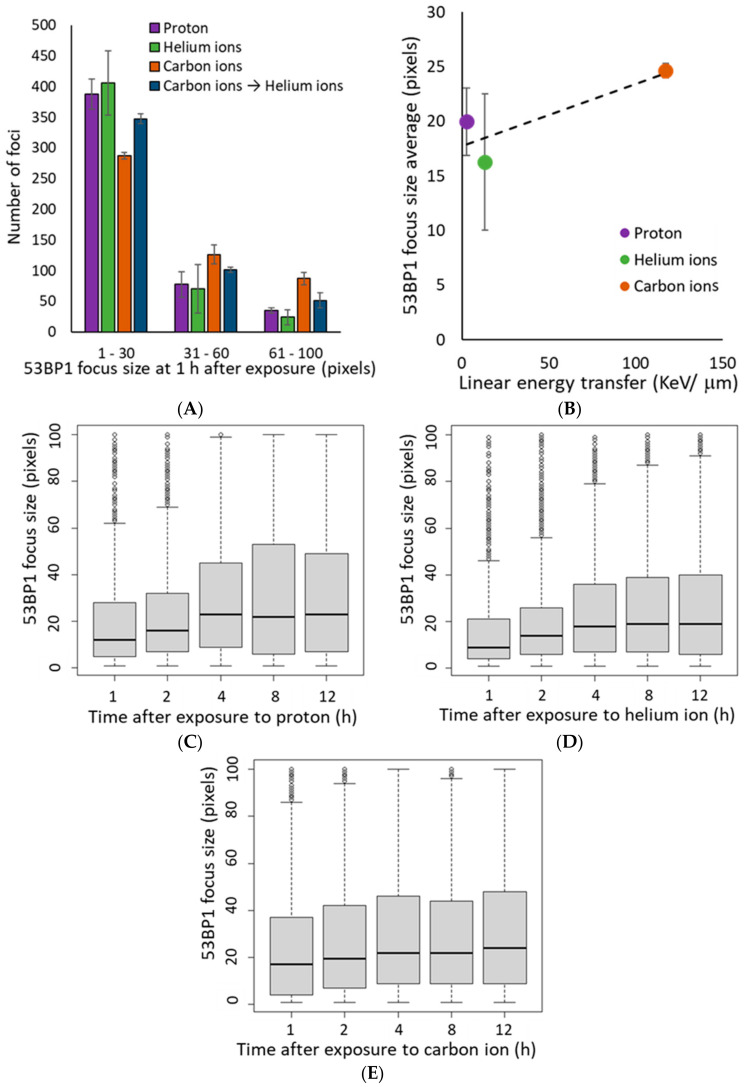
(**A**) The histogram shows the number of 53BP1 foci defined by size at 1 h after irradiation. Foci size indicates the number of pixels calculated using image J for 500 53BP1 foci in each independent experiment (Kruskal–Wallis test of proton vs. helium ions vs. carbon ions vs. carbon ions→helium ion: *p*-value < 2.2 × 10^−16^). (**B**) The mean of average 53BP1 focus size plotted by LET at 1 h after exposure. The dashed line shows the approximate line (R^2^ = 0.7336). The LET 2.73 keV/μm for protons, 13 keV/μm for helium ions, and 117 keV/μm for carbon ions (protons: *n* = 2, helium ions, carbon ions, and mixed carbon ions and helium ions: *n* = 3, respectively. Error bars: SD). (**C**–**E**) The relationship between time after irradiation and change in the size of the 53BP1 foci is shown as a boxplot: (**C**) protons, (**D**) helium ions, (**E**) carbon ions. In boxplot, first quartile to third quartile is represented as a box, and the horizontal bar inside the box is the middle of the dataset. The vertical bars extending up and down from the box represent the maximum and minimum values included in the 1.5 times the IQR, and the dots represent outliers that are not included in the 1.5 times the IQR.

**Figure 5:**
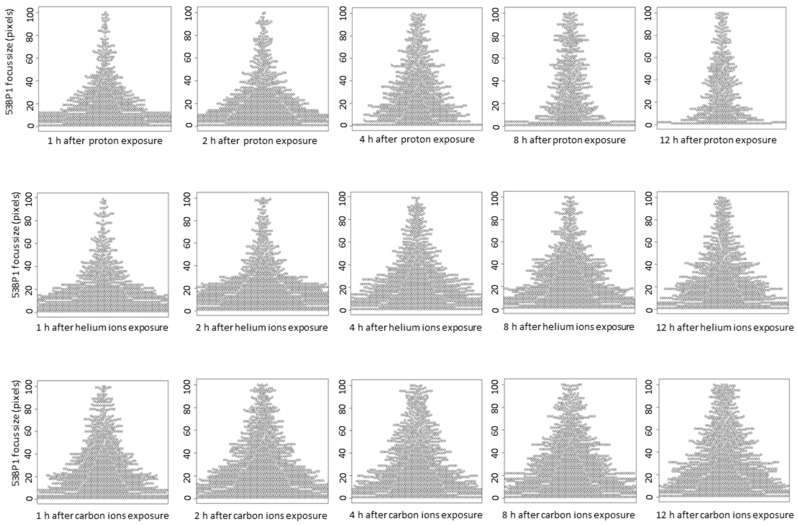
The relationship between time after irradiation and change in the size of 53BP1 foci is shown as a beeswarm plot. In the beeswarm plot, each focus is plotted as a dot to avoid overlapping for each size.

The 53BP1 focus size at 1 h after irradiation increased in an LET-dependent manner, similar to that of γ-H2AX (Figure 4A,B). Interestingly, in the proton and helium irradiated groups, the mean 53BP1 focus size was slightly increased from 1–4 h after irradiation (Figure 4C,D). On the contrary, in the carbon ion-irradiated group, the average focus size of 53BP1 at 1 h after irradiation was comparable to that at 4 h after proton and helium irradiation, and there was a slight increase in focus size with time (Figure 4E). Moreover, detailed analysis of the 53BP1 focus population showed that small 53BP1 foci decreased and large foci increased with time after irradiation (Figure 5). These results suggest that, unlike the γ-H2AX foci, the large 53BP1 foci are likely to remain, whereas small 53BP1 foci decrease rapidly or expand in size.

Moreover, the mixed irradiated samples had an average 53BP1 focus number and size approximately equal to that of the carbon and helium ion irradiated samples at any time point, where no combined effect of mixed irradiation was observed, similar to the trend observed with the γ-H2AX foci (Figure 3 and Figure 4).

## 4. Discussion

Cluster DNA damage is defined as multiple DNA damage within 1–2 helix turns of a DNA molecule [4]. Repair of clustered DNA damage is known to be difficult [13,32], and in fact, the results of this study suggest that the DSB repair kinetics of cells irradiated with high LET carbon ions were significantly slower than those of cells irradiated with protons or helium ions (Figure 1). These results clearly demonstrate the difficulty of repairing DNA damage induced by carbon ions with high LETs that are expected to induce more clustered DNA damage. The size of the γ-H2AX foci associated with DNA damage caused by high LET heavy ions is reportedly larger than that of the γ-H2AX foci associated with X-rays and γ-rays. In this study, we were able to clearly demonstrate the relationship between LET and γ-H2AX foci size, which was not experimentally elucidated earlier. We consider that large γ-H2AX foci have been reported to include multiple foci of RPA that coats the single-stranded DNA at the end of DSBs [30], indicating the presence of multiple DSBs in a large γ-H2AX focus. Notably, cluster DNA damage occurs at the nanometer scale, and therefore cannot be observed directly using the microscopes available currently. However, the large γ-H2AX foci identified in this study would be expected to contain multiple DSBs and some cluster DNA damage, and are considered more complex than the small γ-H2AX foci. Importantly, this study revealed that the large γ-H2AX foci decreased in size and/or number with time, and that not all large γ-H2AX foci remained (Figure 1 and Figure 2). This suggests a gradual dephosphorylation of γ-H2AX as DSB repair progresses in large γ-H2AX foci, although it is unclear whether this is exact repair.

Unlike the γ-H2AX foci, the population of large 53BP1 foci increased after 4 h of irradiation. 53BP1 is known to regulate c-NHEJ progression and DNA end resection, and plays an important role in pathway switching for DSB repair [22,33,34,35]. Ionizing radiation-induced DSBs are repaired through biphasic kinetics involving fast and slow processes [36,37]. Fast processes involve repair by DSB end resection-independent NHEJ, and in slow repair, such as DSB repair in heterochromatin regions or cluster DNA damage, BRCA1 activity results in DSB end resection [19,38,39]. In the S/G2 phase, DSB end resection continues to HR, whereas DSB repair occurs through the slow process of DSB end resection-dependent NHEJ in the G1 phase [36,40,41,42]. Microscopically, 53BP1 repositioning for DSB end resection associated with BRCA1 activation is reported to result in a larger focal size at 2 h post-irradiation than immediately after irradiation using ionizing radiation [17]. Considering the similar function of 53BP1 and BRCA1 in the G1 phase as in the G2 phase [19,40], it is not surprising that the increased focus size of 53BP1 seen in the G2 phase is also visible in the G1 phase. It is also assumed that a large proportion of the cells were irradiated during the G1 phase because this study used normal fibroblasts rather than cancer cells. An increase in the population of large 53BP1 foci was observed after 4 h after irradiation, and the biphasic kinetics switched to slower kinetics (Figure 4 and Figure 5). For all these reasons, it is reasonable to assume that the larger 53BP1 focus size represents repair by the end resection-dependent NHEJ.

Although the average 53BP1 focus size at 1 h after carbon ion irradiation is as large as that at 4 h after proton and helium ion irradiation, it is unclear whether all large foci at 1 h after irradiation with carbon ions represent the end resection-dependent NHEJ. However, the number of 53BP1 foci remained more than 90% at 4 h and 80% at 8 h after carbon ion irradiation, suggesting that the DSBs characterized by large 53BP1 foci are mostly repaired through a slow process. Further data are needed to support the above hypothesis, as our present study only included focus analysis of γ-H2AX and 53BP1. To emphasize our hypothesis, additional experiments which neutral comet assays for validation of the kinetics of DSB rejoining, cell cycle assays for identify the repair pathways and staining for other markers such as RPA for assessment of end resection are required.

In this study, we irradiated cells with carbon ions followed by helium ions at very short intervals and analyzed for the DSB repair of cells irradiated with a mixture of different ion beams. This study is the first to report the combined effect of different types of radiation on DSB repair response and that the type of radiation may not affect the rate of DSB repair characterized by γ-H2AX and 53BP1 (Figure 1 and Figure 3). However, because cytotoxicity and other effects of combined irradiation were not evaluated in this study, the possibility that combined irradiation may affect other aspects of DSB repair dynamics should not be excluded. Other groups have reported that the frequency of chromosomal aberrations differed depending on the interval between proton irradiation and iron irradiation [43]. Therefore, the combined effects of mixed irradiation may affect the accuracy rather than the kinetics of DSB repair.

The LET-dependent changes in the focal size of γ-H2AX and 53BP1 observed in this study may be useful for future biological assessment in astronauts. The results of the present study are expected to provide the radiation dose based on the number of γ-H2AX foci, and information on the radiation species and energy based on γ-H2AX and 53BP1 focus size using lymphocytes from astronauts. However, γ-H2AX and 53BP1 focus assays have several limitations: (1) γ-H2AX and 53BP1 foci decrease with repair, and acute irradiation of cells with 1 Gy of x-ray or γ-ray causes the γ-H2AX and 53BP1 foci levels to decline to background levels after 24 h. Therefore, these are not long-term preserved dose markers. (2) As exposure in space is low-dose-rate chronic [14,27], the amount of DSB produced is very low, and it is likely that the total exposure dose cannot be assessed due to the loss of focus associated with repair, as mentioned in (1). However, DSBs induced by high-LET particle beams should be detectable using a γ-H2AX focus on the track. In fact, Ohnishi et al. reported that clear tracks of the γ-H2AX signals are detected in lymphoblastoid nuclei after spaceflight [44]. Therefore, although the current techniques are difficult to implement, we believe both γ-H2AX and 53BP1 might be future useful biomarkers for assessment of biological effects in astronauts.

## 5. Conclusions

Our data clearly show an increase in complex DNA damage and a delay in the rate of repair dependent on the LET of radiation. Complex DNA damage characterized by the large 53BP1 foci was presumed to be repaired through a slow process involving the end resection-dependent NHEJ, which is more likely to trigger chromosomal translocation. Although radiation sources and exposure dose used in this study may not completely simulate real radiation exposure in space, the results of this study suggest that the risk of space radiation may be greatly influenced by the radiation dose as well as the type of radiation exposure.
